# UNIFESP Info Plastica – An informative application covering the most frequently performed plastic surgeries worldwide

**DOI:** 10.6061/clinics/2018/e244

**Published:** 2018-06-19

**Authors:** José da Conceição Carvalho-Júnior, Alessandra Haddad, Lydia Masako Ferreira

**Affiliations:** Divisao de Cirurgia Plastica, Universidade Federal de Sao Paulo, Sao Paulo, SP, BR

**Keywords:** Plastic Surgery, Information Technology, Software

## Abstract

**OBJECTIVES:**

The objective of this study was to develop a free smartphone application with reliable and useful information for the lay public on the most frequently performed plastic surgeries worldwide.

**METHODS:**

The five most frequently performed surgeries worldwide according to ISAPS (International Society of Aesthetic Plastic Surgery) were selected. Information from the websites of the American Society of Plastic Surgeons (ASPS), American Society for Aesthetic Plastic Surgery (ASAPS) and Brazilian Society of Plastic Surgery (SBCP) and from the Outpatient and Hospital Medicine Guide of the Plastic Surgery Division at the Federal University of São Paulo (UNIFESP) was used for content assembly. YouTube videos with patients’ postoperative testimonials were used for an improved understanding of their real concerns. Printed text was distributed to patients in Brazil’s Unified Health Service to improve comprehensibility. Content on each of the five surgeries was presented in the application with the following layout: What is the surgery?; Who are the candidates?; Preparations for surgery; Stages of surgery; Recovery after surgery; Complications; Choice of surgeon; Consultation; What is the cost? and Glossary. All material was delivered to an outsourced company to produce the application software.

**RESULTS:**

The result was the creation of an application with extensive content on the most frequently performed plastic surgeries.

**CONCLUSION:**

The UNIFESP Info Plastica application is an academically based, free and reliable source of information for the lay public interested in all aspects of the most frequently performed plastic surgeries worldwide.

## INTRODUCTION

The number of plastic surgeries increases every year. According to data provided by the International Society of Aesthetic Plastic Surgery (ISAPS), 9,641,253 aesthetic plastic surgeries were performed worldwide in 2015. Brazil is second in the number of plastic surgeries performed per country, behind only the US, which leads the way in terms of the number of both surgical and non-surgical aesthetic procedures 1.

Considering these data, an increasing number of people are looking for information on plastic surgery. At the same time, it is known that surgical success depends on not only good surgical technique but also patient care during the postoperative period, making this global knowledge essential for patients.

Information dissemination is centered on the Internet, where content can be accessed through computers and laptops and more recently, through portable and practical devices, such as smartphones. In Brazil alone, 55 million smartphones were sold in 2014 2, which represented a record for the country and demonstrated that a significant proportion of the population has adopted this technology. Meanwhile, only 4.5 million computers were sold in 2015, which represented an annual sales decrease of 36%, whereas the average price was 37% higher than in 2014. 3

Brazil has 107.9 million Internet users. In 2016, 93% of them used their cell phone to access the Internet; on the other hand, the percentage of users accessing the Internet by computer decreased from 80% in 2014 to 57% in 2016. 4

Although the Internet is an important source of answers to the questions asked by plastic surgery enthusiasts, in Brazil, where the Internet is expensive, most of the population has difficulty affording access, limiting web use to only places where free Wi-Fi is available.

Regarding Brazilian residences, 54% are connected to the Internet (36.7 million). Access to the web is present in classes A (98%) and B (91%), whereas only 23% of homes in classes D and E are connected to the Internet. 5 One way to access information offline is through applications that once downloaded, can be opened on a smartphone from anywhere at any time without the need to be connected to the Internet.

From the patient’s perspective, the Internet is considered an instructional tool that helps inform decisions about their health.

However, patients seeking aesthetic procedures may be increasingly vulnerable to inappropriate advertising and biased information. Many sites targeting potential patients are driven by financial profit, and they occasionally omit important medical information, such as the risks and complications involved in surgical procedures 6.

Therefore, the need arises to create reliable content with information collected from the websites of major plastic surgery societies about indications and pre- and postoperative care that has adapted for the lay public so that people can better inform themselves before undergoing plastic surgery; this information should be available through a free application that can be accessed from anywhere and at any time.

## METHODS

### Ethics committee

This study was approved by the Research Ethics Committee of the Federal University of São Paulo (UNIFESP) under protocol number 1502290116.

### Priority search

This study was performed in May 2015 and was repeated in May 2016 to update the results. The work entailed app searches on the App Store and Google Play (iOS and Android operating system application download platforms, respectively) using the sites https://itunes.apple.com (App Store) and https://play.google.com/store/apps (Google Play). The following terms/descriptors were used: plastic surgery, aesthetic, reconstructive plastic surgery and cosmetic surgery.

### Assembling the application

The five most performed plastic surgeries worldwide according to the latest ISAPS global data published on their website were chosen and included liposuction, breast augmentation, blepharoplasty, abdominoplasty and rhinoplasty.

The patient information areas of the Brazilian and American Plastic Surgery Societies’ websites were used as information sources when developing the application content about these surgeries.

Information contained on the American Society of Plastic Surgeons (ASPS), American Society for Aesthetic Plastic Surgery (ASAPS) and Brazilian Society of Plastic Surgery (SBCP) websites and in the Outpatient and Hospital Medicine Guide of the UNIFESP Plastic Surgery Division formed the basis of the application’s informative content 7,8,9,10.

Some words were highlighted in blue in the text inside the application to indicate that an explanation could be found in the glossary for these words to improve the user’s understanding of specific surgical terms.

The material was prepared in the following format: What is the procedure?; Who are the candidates?; Preparations for surgery; Stages of surgery; Recovery after surgery; Complications; Choice of surgeon; Consultation; What is the cost? and Glossary. Illustrative figures were also used to facilitate understanding of the content. The planning of this application followed design thinking methodology precepts, which entail a set of methods and processes for resolving problems related to the future acquisition of information for knowledge analysis and the proposal of solutions. The culture, context, personal experiences and processes of individuals’ lives were mapped out to create a more complete view and thus better identify barriers and generate alternatives to overcome them. To this end, design thinking proposes a more empathic view that allows people to be at the center of a project’s development and generate results that are more desirable for them; however, at the same time, these results can be transformed into reality from a technical point of view 11.

Prior to product finalization, a YouTube search was performed to find videos with testimonials given by patients in the postoperative periods of various plastic surgeries to better understand the future user’s wishes and needs. These videos were accompanied by various comments and questions that other Internet users posted for the videos’ authors. The terms used to search the site were as follows: liposuction, breast augmentation, blepharoplasty, abdominoplasty and rhinoplasty 12,13,14,15,16. Fifty videos were evaluated on each type of procedure, and the three most common questions for each type of surgery were recorded. All videos selected for evaluation had the eligibility criteria of being produced by patients who had undergone surgery and who were reporting their experiences during the postoperative period.

After the content was prepared as described above, all material was printed on A4 sulfite paper in the form of plain text. This material was delivered to five patients from each outpatient clinic of the UNIFESP Plastic Surgery Division who were in the preoperative period for the specific surgery upon which the informative material was based.

All participants signed informed consent forms. The selected patients met the following inclusion criteria: adult (>18 years), literate, not having had psychiatric treatment, treated at the UNIFESP Plastic Surgery Division clinic and being in the preoperative phase of treatment. The non-inclusion criteria were the following: patients who refused to read the content and those who did not sign the consent form.

The patients read the informative material and marked the terms or parts that they did not understand with a pen. A reserved space was left at the end of the printed material so that patients could raise questions or suggest information not included in the text. A co-creation process was thus established in which the patient helped develop and improve the product, ultimately leading to more intelligible and useful material for the lay public.

After this patient interaction phase, all content was reviewed by the coordinators of sections corresponding to the surgeries listed in the UNIFESP Plastic Surgery Division. After review, the material was passed on to the outsourced company (DOUGLAEVARISTO Web Soluções, Goiânia, GO, Brazil) to develop the software platform and the www.infoplastica.com.br website with content guidance provided by the authors of this study.

## RESULTS

### Priority search

**Figure 1:**
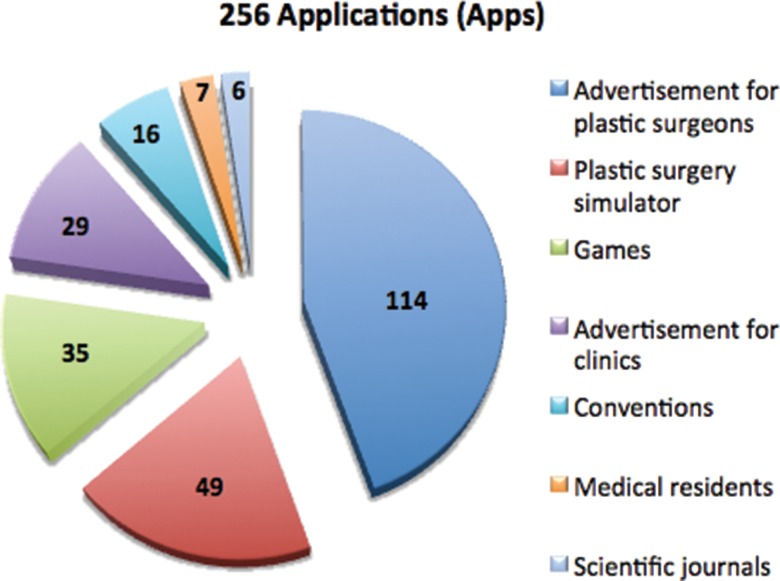
Applications found in the App Store.

In total, 256 results were found in the App Store and 250 were found in Google Play, all of which were contained in the 256 results found in the App Store. No applications were found that was exclusively informative and aimed at the lay public nor were any applications in which a university or plastic surgery service provided this type of information (Figure 1).

### Content

**Figure 2:**
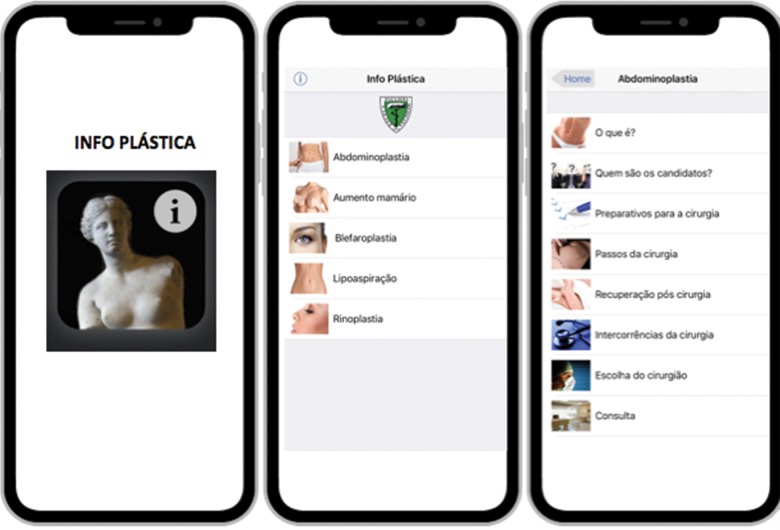
App icon and home screen showing the procedures and their corresponding topics.

The Info Plastica application is available on both the App Store and Google Play without cost. The download links can also be accessed through the www.infoplastica.com.br site (Figure 2).

### Testimonials

Evaluations of the comments and questions asked by Internet users on the 50 postoperative videos for each surgery led to detection of the most frequent concerns. A summary of the three most frequent questions specific to each type of procedure is described in Table 1.

### Co-creation

Twenty-five outpatient clinic patients read the application content. The words that generated the most doubts and that were the most highlighted were the following: diastase (60%), ectropion (56%), blepharoplasty (48%), transconjunctival incision (48%), mastopexy (40%), asymmetry (40%), columella (36%), ecchymosis (32%), septum (28%), seroma (24%), capsular contracture (24%), hematoma (20%), suture (16%), areola (12%) and edema (4%).

## DISCUSSION

An increasing number of people are accessing websites in search of health knowledge 17. In the US, 74% of adults use the Internet with 8 of 10 users seeking medical information through online content 18,19. Among these users, 70% report that the information found affects their treatment decision 10. In Brazil, a study showed that 44.7% of participants had performed an Internet search relating to their child’s health 21. Regarding plastic surgery, it has been observed that 34% of sites related to the “breast augmentation” topic presented false or misleading information about the surgical technique, benefits and risks 22. This lack of reliable online literature led the authors to create an application that would provide reliable information to the lay public.

Considering the discrepancy in Internet access between different economic classes in Brazil and an intention to democratize knowledge, we provided an option to make the content available through an application. The advantages of this feature include easy, free download and offline access.

Brazil is the second largest market for downloads on Google Play and the App Store, and 84% of consumers use up to 10 apps per day and an average of 29 apps per month. Engagement, that is, the number of times an application is opened, is 53.62 times per month. These data demonstrate the incorporation of this technology in Brazilian daily life and confirm applications as a tool of great utility. 23

Information provided by the three largest plastic surgery societies in the world and by the Plastic Surgery Manual from UNIFESP, where the study was performed, were used to guarantee content reliability. In contrast, 69% of the plastic surgeon advertisement applications were devoid of references regarding their available content. In the US, the ASPS has created a standard application that can be purchased by associated surgeons and used for advertising. This app contains areas for summary information about procedures, a glossary, a surgery simulator and an area to introduce the surgeon. All information is approved by the ASPS. However, this app does not show information regarding risks, pre- and postoperative care or possible complications 24.

The YouTube search provided information on what aspects might be interesting to include in the application. During this research on YouTube, the precarious nature of information available on the Internet became even clearer. Many people try to resolve their concerns by watching videos, some of which had more than 1.5 million views; these videos were made by newly operated lay patients who were willing to offer often inadequate guidance on “what to expect during the postoperative period” 17,25. This finding further reinforced the validity of this application as an instructional tool.

Special attention was given to the level of complexity of the language used to make the content more intelligible to readers with lower levels of education by adjusting words misunderstood by outpatients and creating a glossary. The words highlighted in the glossary can serve as a warning to surgeons and serve to highlight the difficulty that patients encounter in understanding their doctors during consultations. Souza et al. 26 created an application for dental care patients with special needs and noted the need for a more intelligible language. A patient’s knowledge of health is the most important individual predictor of their involvement and satisfaction with the final results of medical treatment 13. Helping the population gain access to a clearer and more serious view of the world of plastic surgery was the driving force behind this application.

With the growth in the number of plastic surgeries performed, many patients with lower purchasing power could benefit from these surgeries. This population has for the most part, a comparatively low educational level and therefore has little access to information regarding the procedures performed. This application offers the capacity to disseminate this information in a correct, intelligible and free manner. Other applications, such as that developed by Oliveira and Costa 27, also aimed to deliver free information that was intelligible even to users with lower educational levels.

As a limitation, the Info Plastica UNIFESP presents information on only the five most performed plastic surgeries worldwide, representing 56% of all surgeries 1. That is, this partial version of the application does not contemplate all surgical modalities within the specialty. In addition, despite growth in the use of applications through the incorporation of smartphones, a portion of the population does not have access to the content of the application either because they do not have a smartphone (32% of the population) 5 or because they use any operating system other than iOS or Android, which are present in 99.1% of all smartphones 23.

During the writing of this article, the application was available for download from the App Store and Google Play, and the numbers reinforce its relevance: it was the sixth most downloaded application in Brazil when searching for the term plastic surgery on the two platforms. This result was obtained without any marketing campaign, which demonstrates the lack of similar tools and the enormous potential of this application.

The UNIFESP Info Plastica application is an academically based, reliable, and free source of information for the lay public interested in all aspects of the most frequently performed plastic surgeries worldwide.

## AUTHOR CONTRIBUTIONS

Carvalho-Júnior JC conceived the original idea and was responsible for the application content development. Haddad A contributed to the original idea and was responsible for the review of the manuscript. Ferreira LM contributed to the original idea, provided orientation during the research development and was responsible for the review of the manuscript.

## Figures and Tables

**Table 1:** Results for the Three Most Frequently Asked Questions among YouTube Users for each Surgery.

**Liposuction**
1- Lymphatic drainage. How long after surgery?
2- For how long must one wear a compression garment?
3- How many pounds are lost in surgery?
**Breast Augmentation**
1- What is the best way to place the implant? Axillary? Inframammary? Periareolar?
2- What is the best implant position? Under or over the muscle?
3- Is it necessary to change the implant? After how long?
**Blepharoplasty**
1- For how long do eyelids remain swollen?
2- Can a smoker have this surgery? How long before surgery should I stop smoking? How long afterwards can I return to smoking?
3- Cold compresses on the eyelids. For how long? How often?
**Abdominoplasty**
1- How is the postoperative period? (Pain, Drainage Care)
2- Do I need to lose weight to undergo the surgery?
3- What is the final appearance of the abdominal scars?
**Rhinoplasty**
1- Why is there a purple area under the eyes?
2- How long is the hospital stay?
3- What type of anesthesia is used in the surgery?
